# Surgical Needs of Refugee Populations in the European Union: Implications for Plastic and Reconstructive Surgery

**Published:** 2016-09

**Authors:** Peter Niclas Broer, Sabrina Juran

**Affiliations:** 1 Department of Plastic Surgery, Klinikum Bogenhausen Teaching Hospital, Technical University Munich, Germany;; 2 Data and Population Analysis Department, United Nations Population Fund, Technical Division, Population and Development Branch, United Nations, New York, NY, USA

**Keywords:** Global surgery, Global burden of disease, Plastic surgery, Refugee

In 2015, hundreds of thousands of refugees decided to take up the dangerous and oftentimes deadly crossing into countries of the European Union (EU). Many of them were already living in dismal situations. Millions have been trapped. This situation deteriorated throughout the year. Due to the war in Syria, but also other humanitarian situations and developmental crises, including harsh poverty and social deprivation, the EU is currently facing a very high inflow of refugees and other migrants, with many children among them.^1^^,^^2^

Civil unrest and armed conflicts generate vital and complex needs in emergency medicine, surgery, and rehabilitation. Health conditions of refugee populations vary greatly based on origin, geographic location, underlying medical, and nutritional status, and the events causing the initial relocation. The humanitarian condition of refugees can have progressed over a long time. The years in the conflict region or refugee camps might have marked them with relative malnutrition, and insufficient access to medical services so that some may present with diseases which are uncommon under normal conditions. As the humanitarian crisis progresses, malnutrition and disease prevalence increases making a population more susceptible to illness with compromised immune systems and poorer wound healing mechanisms. During a crisis situation, resources are limited and the utilization of expensive resources for patients that will not survive is unwarranted and wasteful.^1^^,^^2^

While recent action on surgical care for refugees tended to focus on the acute phase of the crisis situations, this paper posits that a substantial burden of non-acute morbidity amenable to surgical intervention among refugees and other migrants upon arrivals falls upon the host countries, and their societies. Drawing on past experience in humanitarian crisis situations is crucial to understand the complexity of treating patients presenting with injuries with a long time lag, as not only humanitarian conditions prevent patients from seeking surgical treatment, but also financial, structural as well as cultural barriers exist.^3^

EU Member States are challenged with the medical needs of refugees upon arrival, in particular surgical needs. Large inflows of people overstretch health services available to refugees and to others of the affected countries. Although infectious diseases, malnutrition and diarrhea account for the vast majority of deaths in many crisis situations, many individuals suffer from traumatic injuries and other surgically treatable conditions.^4^

These challenges could be addressed when leaving the beaten track and implementing pragmatic procedures. To understand the determinants involved for surgical interventions, a public health approach is necessary, which could be facilitated by an assessment during the emergent and chronic phases of refuge. In order to improve the lives of refugee populations, successful interventions need to be identified and build upon for similar conditions that affect people in different settings.^5^

Assessing the demand and needs for surgical treatment of refugees will prevent long lasting disadvantages resulting from chronic disease or malfunction. Because many refugees will experience adverse outcomes during the resettlement period, it is essential that the health screenings be performed at first point of arrival as well as during each primary care visit. Hermasson et al displayed the impact of injuries as a result of war and conflict among war-wounded refugees during hospitalization shortly after arrival, and after two years on physical disability as well as wellbeing, and social integration in refugees in Sweden. Primary types of injuries that required surgical intervention were: fractures, traumatic amputations, spinal cord injuries, nerve injuries, combinations of fractures and nerve injuries, bilateral eye injuries, brain injuries, other injuries.^6^

In most cases today, quantitative as well as qualitative data regarding the expected surgical need do not exist. It is hence crucial to collect that kind of information upon arrival to develop a systemic understanding of refugees’ needs and thus be in a position to address these. First-aid posts need to be established in transit areas and refugee camps with an inter-disciplinary team of physicians. In refugee camps or reception centers, with a large population of refugees, the patient volume, the severity of the injury as well as the number and expertise of the medical staff available need to be considered when planning for medical care.^7^

Since financial and human resources are limited, treatment needs must be well established. In Germany, which is currently accepting a high percentage of all refugees, the authorities of the respective federal state are responsible for the medical care as long as refugees remain in the initial reception centers. After a few weeks, refugees receive a treatment card with which they can receive services in the practices of private physicians. For treatment of complex injuries, for instance severe burn contractures or injuries of the extremities requiring reconstruction, a statement of need including the estimated length of treatment and overall treatment cost has to be filed by the treating surgeon, which in turn has to be reviewed and granted by the respective local state authorities where the refugee is currently located.^7^

Communication with the refugees can present a large burden in this context. Among many refugees however, there are numerous highly skilled physicians and other health professionals that need to be identify to support the efforts of the host country- through interpretation as well as by potentially supporting medical treatment under the supervision of national physicians. Further, private prescriptions for medications in the name of an asylum seeker need to be collected, with pharmacies providing the collective centers on a daily basis. In collaboration with social security vouchers could advance the pharmacies’ expenses. However not only language and financial barriers, but also gender, and cultural issues need to be considered when treating refugee patients.^7^

In conclusion, past experience has shown that refugee situations bring along a considerable burden of non-acute surgical morbidity. The capacity of EU Member States under particular migratory pressure needs to be strengthened to provide the arriving refugees with surgical assessment and where possible referrals for treatment, thus contributing to addressing the burden of global surgical disease. Estimating surgical needs up front upon arrival at refugee camps or reception centers can act as a cost-effective way to relieve some of this burden.^8^ Plastic surgeons have the unique opportunity to make a difference in the lives of refugees because they play a pivotal role in the assessment, screening, and referral of refugees for surgical treatment, and providing support to resettlement countries to address the broad needs of their refugee populations.

Ethical Standards

The manuscript does not contain clinical studies or patient data.

## CONFLICT OF INTEREST

The authors declare no conflict of interest.
